# Mechanical Power and Acute Kidney Injury in Mechanically Ventilated Critically Ill Patients: A Single-Center Retrospective Cohort Study

**DOI:** 10.3390/medicina62101868

**Published:** 2026-09-27

**Authors:** Zafer Çukurova, Dicle Birtane, Mehmet Süleyman Sabaz

**Affiliations:** 1 Department of Anesthesiology and Reanimation, Intensive Care, Bakirkoy Dr. Sadi Konuk Training and Research Hospital, Istanbul 34147, Turkey; dicle1tane@gmail.com; 2 Department of Anesthesiology and Reanimation, Intensive Care, Marmara University Pendik Training and Research Hospital, Istanbul 34899, Turkey; udmss_47@hotmail.com

**Keywords:** acute kidney injury, invasive mechanical ventilation, mechanical power

## Abstract

*Background and Objectives*: Acute kidney injury (AKI) is highly prevalent among critically ill patients receiving invasive mechanical ventilation (IMV) and is associated with adverse outcomes. Mechanical power (MP) reflects the total energy delivered to the respiratory system, but its association with AKI in mechanically ventilated critically ill patients remains incompletely understood. Few studies have examined the relationship between MP and AKI development. *Materials and Methods*: Adult patients with MP data available for each of the first five consecutive days of IMV constituted the study population. MP and ventilatory parameters were recorded during this period. Factors independently associated with AKI were identified using multivariable logistic regression. The discriminative performance of MP for AKI and continuous renal replacement therapy (CRRT) use was evaluated. *Results*: Among 924 patients, 605 (65.5%) developed AKI. MP was consistently higher in patients who developed AKI during the first five days of IMV (11.45 ± 2.88 vs. 9.48 ± 2.50 J/min; *p* < 0.001). Although PEEP, peak inspiratory pressure, driving pressure, respiratory rate, and FiO_2_ were higher in the AKI group, tidal volume and respiratory system compliance did not differ significantly. In multivariable analysis, higher mean MP (OR = 1.330, 95% CI 1.235–1.432; *p* < 0.001), greater mean fluid balance (OR = 1.477, 95% CI 1.225–1.780; *p* < 0.001), older age, and trauma were independently associated with AKI. MP showed moderate discrimination for AKI (AUC = 0.681), with an optimal cutoff of 10.38 J/min. For CRRT use, MP showed significant but limited discrimination (AUC = 0.624; 95% CI, 0.584–0.664; *p* < 0.001). *Conclusions*: In critically ill patients receiving IMV, higher MP remained associated with AKI after adjustment for measured covariates. The 10.38 J/min threshold is hypothesis-generating and requires external validation.

## 1. Introduction

Although mechanical ventilation (MV) is a life-saving therapy for critically ill patients with acute respiratory failure, its effects are not limited to the lungs alone. Hemodynamic alterations induced by positive-pressure ventilation, impaired gas exchange, and the systemic inflammatory response may adversely affect the function of other organs, particularly the kidneys. Acute kidney injury (AKI) observed during invasive mechanical ventilation (IMV) is associated with increased intensive care unit (ICU) mortality, prolonged ventilator dependence, longer ICU length of stay (LOS), and impaired respiratory mechanics [1]. These extrapulmonary effects of MV highlight the clinical relevance of organ crosstalk in critically ill patients, particularly the interaction between pulmonary and renal dysfunction [2,3,4]. Indeed, the incidence of AKI is known to increase in patients receiving IMV [5,6].

There is a bidirectional and dynamic interaction between the lungs and kidneys. AKI may adversely affect pulmonary function by leading to fluid overload, metabolic acidosis, accumulation of uremic toxins, and release of inflammatory mediators. These changes may disrupt the endothelial–epithelial barrier of the lung. Conversely, acute lung injury and MV can also lead to renal injury via various pathways related to inflammation (biotrauma), hemodynamic alterations, and gas exchange. Together, these mechanisms may amplify dysfunction across both organ systems in critically ill patients [6,7,8].

It remains unclear which component of MV plays a more important role in kidney injury. When traditional ventilator parameters such as tidal volume (TV), driving pressure (DP) or positive end-expiratory pressure (PEEP) are assessed independently, they evaluate only a single component of MV. In contrast, mechanical power (MP) integrates TV, respiratory rate (RR), airway pressure and flow into a single variable, thereby representing the total amount of energy transferred from the ventilator to the respiratory system [9,10]. In our study, five-day mean MP remained associated with AKI after adjustment for PEEP, TV, disease severity, and other measured clinical variables. Higher MP has been associated with poor clinical outcomes in various groups of critically ill patients [11,12,13,14]. However, evidence regarding the relationship between MP and AKI remains limited. An experimental study showed that individual components of MP may affect the kidneys differently, with the PEEP component showing the strongest association with AKI [15]. To date, clinical studies have predominantly focused on individual ventilatory parameters, such as TV, PEEP, or DP. In contrast, clinical evidence evaluating the association between MP and AKI remains scarce. Whether the integrated mechanical energy load represented by MP is associated with clinically relevant renal outcomes therefore warrants further investigation.

To address this gap, we evaluated MP and renal outcomes in a large clinical cohort of mechanically ventilated critically ill patients. This study aimed to evaluate the association between MP and AKI development in critically ill adult patients receiving IMV and to assess the discriminative performance of MP for AKI and continuous renal replacement therapy (CRRT) use. By evaluating both AKI development and the requirement for CRRT, we sought to characterize the relationship between global ventilatory energy load and clinically relevant renal outcomes. Patients who developed AKI were also compared with those who did not in terms of ventilatory parameters, respiratory mechanics and clinical outcomes.

## 2. Materials and Methods

### 2.1. Ethical Considerations

The study was approved by the Clinical Research Ethics Committee of Bakırköy Dr. Sadi Konuk Training and Research Hospital (Protocol Code: 2025/203; Decision No.: 2025-13-14; Date: 23 July 2025) and was conducted in accordance with the principles of the Declaration of Helsinki.

### 2.2. Study Design and Setting

This retrospective cohort study included clinical data from patients admitted to a tertiary adult ICU at a training and research hospital in Istanbul, Türkiye, between 1 January 2015 and 1 January 2025. Ethical approval was obtained on 23 July 2025, before any study-specific data extraction or analysis was initiated. The ICU operates as a closed unit with a nurse-to-patient ratio of 1:2 and provides 24/7 specialist-led critical care by anesthesiology and reanimation specialists, intensive care specialists, and residents. The unit is supported by a clinical decision support system and has round-the-clock capability to provide extracorporeal therapies, including extracorporeal membrane oxygenation (ECMO), CRRT, and plasma exchange.

#### 2.2.1. Study Population

All adult patients admitted to the ICU during the study period were screened using the electronic medical record system. Patients aged <18 years, pregnant patients, those with chronic kidney disease (CKD) or AKI clinically diagnosed before or at ICU admission and documented as an ICU admission diagnosis were excluded. Additional exclusion criteria were major respiratory comorbidities (including home oxygen therapy or stage 4 chronic obstructive pulmonary disease [COPD]), those with major thoracic trauma, IMV for fewer than five consecutive days, and incomplete mechanical power (MP) data during the first five consecutive days of IMV. The five-day observation period was selected to provide a standardized window for assessing sustained ventilatory exposure and temporal changes in MP rather than relying on a single measurement. This approach required patients to remain invasively ventilated throughout the observation period and therefore excluded patients who were extubated, died, had interruption of IMV, or had incomplete ventilatory data before completion of five consecutive days. Patients aged ≥18 years with MP data available in the electronic medical record for each of the first five consecutive days of IMV and who received IMV throughout this period constituted the final study population.

Among these patients, AKI status was assessed daily from ICU admission onward according to the Kidney Disease: Improving Global Outcomes (KDIGO) criteria using both serum creatinine and urine output criteria. The first serum creatinine value measured at ICU admission was used as the baseline creatinine because reliable pre-ICU creatinine measurements were not consistently available for all patients. New-onset AKI during the subsequent five-day observation period was identified based on changes in serum creatinine relative to this baseline value together with the KDIGO urine output criteria. Hourly urine output was routinely recorded by nursing staff in the electronic medical record system, indexed to ideal body weight, and evaluated over the KDIGO-defined time intervals. Diuretic use was not incorporated into the AKI classification. Patients receiving CRRT were classified as KDIGO stage 3. AKI developing within the first 24 h after ICU admission was considered incident AKI during follow-up. AKI status was assessed daily, and the maximum AKI stage reached during the observation period was recorded. Patients with missing data required for AKI assessment were excluded. Patients were subsequently categorized into two groups according to whether they developed AKI during follow-up: those who developed AKI and those who did not (Figure 1).

#### 2.2.2. Data Collection

After eligible patients were identified, data were extracted from the hospital electronic medical record system and the clinical decision support system used in the ICU (EMRall-QlinICU, IMDSoft MetaVision, V.3.46.38, Tel Aviv, Israel) using Structured Query Language (SQL) queries. Demographic characteristics, comorbidities, ICU admission diagnoses, and clinical and laboratory data at ICU admission were recorded. Pulmonary cause of ICU admission was recorded as an admission diagnosis category and did not represent pre-existing chronic pulmonary disease. Disease severity was assessed at ICU admission using the Acute Physiology and Chronic Health Evaluation II (APACHE II) and Sequential Organ Failure Assessment (SOFA) scores, while neurological status was evaluated using the Glasgow Coma Scale (GCS). Vital signs, laboratory variables, MV parameters, and MP measurements were recorded during the first five days of IMV. The five-day observation period was selected to characterize sustained ventilatory exposure and temporal changes in MP rather than relying on a single measurement. Patients who were extubated, died, had interruption of IMV, or had incomplete ventilatory data before completing five consecutive days were therefore excluded. TV was normalized to predicted body weight (PBW), calculated according to patients’ height and sex, and was expressed as mL/kg PBW. Lung-protective ventilation strategies were part of routine clinical practice at our center. Ventilator settings were individualized according to the patient’s clinical condition and respiratory-system mechanics, with the aim of using low TV and limiting airway pressures whenever clinically feasible. Finally, the duration of IMV, ICU LOS, and ICU mortality were recorded for all patients.

#### 2.2.3. Mechanical Power Calculation

IMV was delivered using Maquet Servo-i/u ventilators (Getinge, Solna, Sweden). Of the 924 patients, 835 (90.4%) were ventilated using pressure-controlled ventilation (PCV) and 89 (9.6%) using volume-controlled ventilation (VCV). No transitions between these ventilation categories occurred during the five-day observation period. MP was automatically calculated using the corresponding mode-specific equations. Ventilatory parameters were automatically transferred from the mechanical ventilator to the electronic clinical record system and recorded at one-minute intervals. These minute-by-minute recordings were aggregated by the system into hourly mean values. A minimum of 23 h of ventilator data within each 24-h period was required to calculate a daily MP value, allowing a maximum cumulative interruption of 1 h per day. Hourly values were calculated from the available minute-level measurements within each represented hour, and daily MP was calculated as the mean of the available hourly values. This data-availability criterion was required to be met on each of the five consecutive observation days. Transient interruptions in minute-level data, including those occurring during recruitment maneuvers, airway suctioning, temporary circuit disconnection, or intermittent spontaneous respiratory activity, were accepted only when they did not exceed 1 h. Hourly values were calculated from the available measurements within each represented hour, and daily MP was calculated as the mean of the available hourly values. Daily and five-day mean ventilatory parameters were compared between patients who developed AKI during follow-up and those who did not.

TV was normalized to PBW, calculated according to height and sex, and expressed as mL/kg PBW. Patients with incomplete MP data during the five-day observation period were excluded. Potentially implausible or extreme ventilator values were verified against the electronic medical records; values confirmed as true clinical measurements were retained, whereas erroneous values were excluded. During pressure-controlled ventilation, MP was calculated using the simplified bedside equation: MP = 0.098 × RR × TV × (PEEP + ΔPinsp) [16]. During VCV, MP was calculated using the simplified equation described by Gattinoni et al.: MP = 0.098 × RR × TV × (Ppeak − DP/2) [10]. These simplified bedside equations were selected because they could be implemented directly within the electronic medical record system, allowing MP to be calculated automatically from routinely recorded ventilatory variables. This approach was particularly suitable for the retrospective design of the study.

The method used to obtain plateau pressure differed between ventilation modes. During VCV, a 10% Tpause was routinely applied as the default setting of the Servo-i ventilator. This pause allowed Pplateau to be measured automatically at the end of inspiration, without an additional manual inspiratory hold. Total PEEP was also obtained automatically once expiratory flow had returned to zero. Both Pplateau and total PEEP were transferred to the MetaVision server and stored at one-minute intervals. DP was then calculated automatically as the difference between Pplateau and total PEEP. During PCV, airway pressure was regarded as stable at end-inspiration. Thus, Ppeak was used as an estimate of Pplateau, with Pplateau assumed to equal Ppeak for the retrospective analysis. The same definition of DP (Pplateau − total PEEP) was applied in both VCV and PCV.

#### 2.2.4. Study Outcomes

The primary outcomes were the association between MP and the development of AKI and the discriminative performance of MP for AKI and CRRT use. Secondary outcomes included ICU length of stay, duration of IMV, and mortality.

### 2.3. Statistical Analysis

Statistical analyses were performed using SPSS version 22.0. The normality of continuous variables was assessed using the Kolmogorov–Smirnov and Shapiro–Wilk tests. Categorical variables were expressed as frequencies and percentages. Normally distributed continuous variables were presented as mean ± standard deviation (SD), whereas non-normally distributed variables were reported as median and interquartile range (IQR). Between-group comparisons of continuous variables were performed using the independent-samples *t*-test or the Mann–Whitney *U* test when the assumptions of the parametric test were not met. Categorical variables were compared using the chi-square test or Fisher’s exact test, as appropriate.

The discriminative performance of mean MP for AKI development and CRRT use was evaluated using receiver operating characteristic (ROC) curve analysis and reported as the area under the curve (AUC) with 95% confidence intervals (CIs). Optimal cutoff values were determined using the Youden index, and sensitivity and specificity were calculated for the identified thresholds.

Multivariable logistic regression was performed to identify factors independently associated with AKI. The model included age, APACHE II score, SOFA score, diabetes mellitus, hypertension, sepsis, trauma, mean mechanical power, PEEP, tidal volume/PBW, and mean fluid balance. Covariates were selected based on clinical relevance, biological plausibility, and potential confounding effects rather than solely on univariable *p* values. Multicollinearity was assessed using variance inflation factors (VIFs) and tolerance values. Linearity in the logit for continuous predictors was evaluated using the Box–Tidwell procedure with Bonferroni correction. Model calibration was assessed using the Hosmer–Lemeshow test and agreement between observed and predicted AKI probabilities across deciles of predicted risk. Model discrimination was evaluated using the AUC. The stability of regression coefficients was assessed using 1000 bootstrap resamples with bias-corrected and accelerated (BCa) 95% CIs. No imputation was required because there were no missing data for variables included in the final model. All reported *p* values were two-sided, and *p* < 0.05 was considered statistically significant, except for the Bonferroni-adjusted Box–Tidwell tests. No adjustment for multiple comparisons was applied to the exploratory day-by-day comparisons.

## 3. Results

Of 21,280 ICU admissions screened during the study period, 924 patients met the predefined eligibility criteria and were included in the final analysis. Of these, 605 (65.5%) developed AKI, whereas 319 (34.5%) did not. There were no significant differences between the groups in terms of age, sex, body mass index, APACHE II, SOFA, or GCS scores, comorbidities, or ICU admission diagnoses (all *p* > 0.05). Although diabetes mellitus was more prevalent in the AKI group, the difference did not reach statistical significance in the univariable analysis (21.0% vs. 16.0%; *p* = 0.067) (Table 1).

Regarding MV parameters, PEEP and peak inspiratory pressure were significantly higher in patients who developed AKI than in those who did not from the first day and throughout the entire observation period (all *p* < 0.05). FiO_2_ and mean airway pressure were significantly higher in the AKI group from the second day onward (all *p* < 0.05). The five-day mean FiO_2_ (46.8 ± 10.2% vs. 44.3 ± 8.8%), PEEP (7.78 ± 1.85 vs. 7.29 ± 1.82 cmH_2_O), peak inspiratory pressure [22.0 (19.0–25.7) vs. 21.0 (17.8–23.3) cmH_2_O], and mean airway pressure (13.3 ± 3.0 vs. 12.4 ± 2.9 cmH_2_O) were significantly higher in the AKI group (all *p* < 0.001). In contrast, the five-day mean TV was similar between the AKI and non-AKI groups (7.0 ± 0.99 vs. 6.73 ± 1.92 mL/kg; *p* = 0.081). RR was significantly higher in the AKI group from the third day onward (all *p* < 0.001)**.** Plateau pressure was significantly higher in the AKI group throughout the five-day observation period (Table 2).

When parameters reflecting the energy load of MV were evaluated, mechanical power (MP) was significantly higher in patients who developed AKI on all days of follow-up. The five-day mean MP was 11.45 ± 2.88 J/min in the AKI group compared with 9.48 ± 2.50 J/min in the non-AKI group (*p* < 0.001). Similarly, DP was significantly higher in the AKI group on each study day and for the five-day mean (14.79 ± 3.27 vs. 13.51 ± 2.73 cmH_2_O; *p* < 0.001). Ventilator work of breathing (WOBv) was also higher in the AKI group throughout the observation period [five-day mean: 1.18 (1.05–1.36) vs. 1.08 (0.94–1.26) J/L; *p* < 0.001], whereas no significant between-group difference was observed in respiratory system compliance (*p* = 0.451) (Table 3).

The duration of MV [9.91 (6.70–14.31) vs. 10.58 (7.54–15.54) days; *p* = 0.041] and ICU LOS [12.04 (7.43–18.41) vs. 13.29 (8.75–20.16) days; *p* = 0.014] were shorter in the AKI group. In contrast, both 28-day mortality (48.6% vs. 33.5%; *p* < 0.001) and ICU mortality (50.4% vs. 37.3%; *p* < 0.001) were significantly higher among patients who developed AKI (Table 3).

In the ROC analysis, the discriminative performance of mean MP for distinguishing between patients who developed AKI and those who did not was moderate, with an AUC of 0.681 (95% CI, 0.646–0.716; *p* < 0.001). The optimal cutoff value identified by ROC analysis was 10.38 J/min, yielding a sensitivity of 62.0%, specificity of 61.4%, Youden index of 0.234, and balanced accuracy of 61.7% (Figure 2).

The distribution of AKI stages and renal outcomes among patients who developed AKI was further evaluated according to 28-day survival status (Table 4). The proportion of patients with AKI did not differ significantly between survivors and non-survivors on any of the first five study days (all *p* > 0.05). However, the distribution of AKI stages differed significantly between survivors and non-survivors on Day 3 (*p* < 0.001) and Day 5 (*p* = 0.020). AKI on the last day of ICU stay was significantly more frequent among non-survivors than among survivors (79.3% vs. 56.6%; *p* < 0.001). CRRT use during the five-day observation period was also more frequent among non-survivors (40.5% vs. 20.6%; *p* < 0.001) (Table 4).

In the multivariable logistic regression analysis, higher mean MP, greater mean fluid balance, older age, and trauma were independently associated with AKI. Mean MP remained independently associated with AKI after adjustment for mean fluid balance and the other covariates included in the model (OR = 1.330, 95% CI 1.235–1.432; *p* < 0.001). Each 1-L increase in mean fluid balance was associated with a 47.7% increase in the odds of AKI (OR = 1.477, 95% CI 1.225–1.780; *p* < 0.001). Age (OR = 1.012, 95% CI 1.003–1.022; *p* = 0.009) and trauma (OR = 1.775, 95% CI 1.094–2.879; *p* = 0.020) were also independently associated with AKI. APACHE II score, SOFA score, diabetes mellitus, hypertension, sepsis, PEEP, and tidal volume normalized to PBW were not independently associated with AKI. The model demonstrated adequate calibration (Hosmer–Lemeshow *p* = 0.456) and acceptable discrimination (AUC = 0.705, 95% CI 0.670–0.739), with a Nagelkerke R^2^ of 0.181. Bootstrap analysis with 1000 resamples supported the stability of the main coefficient estimates (Table 5). In a sensitivity analysis excluding patients who developed AKI within the first 24 h, 634 patients were evaluated. Of these, 284 subsequently developed AKI and 350 did not. First-24-h MP was significantly higher in patients who subsequently developed AKI than in those who did not [median (IQR): 10.92 (8.98–13.00) J/min vs. 10.48 (8.00–12.73) J/min; *p* = 0.009]. In a separate sensitivity analysis additionally adjusting for DP and PEEP, mean MP remained independently associated with AKI (OR = 1.364, 95% CI: 1.262–1.474; *p* < 0.001).

The trajectories of renal, metabolic, and hemodynamic variables during the first five days of follow-up are presented in Figure 3. Patients who developed AKI exhibited higher creatinine and urea levels, lower estimated glomerular filtration rate (eGFR), and a more positive fluid balance over the observation period. In addition, lower pH and HCO_3_^−^ levels and higher PaCO_2_ values were observed in the AKI group. Daily trajectories of C-reactive protein (CRP), procalcitonin, and mean arterial pressure (MAP) were also compared between the groups (Figure 3).

The discriminative performance of five-day mean MP for CRRT use was evaluated using ROC analysis. The AUC was 0.624 (95% CI, 0.584–0.664; *p* < 0.001), indicating statistically significant but limited discriminative performance. The optimal cutoff value determined by the Youden index was 10.39 J/min, with a sensitivity of 69.9% and a specificity of 51.6% (Figure 4).

## 4. Discussion

In this retrospective cohort study of critically ill patients with comparable baseline disease severity, patients who developed AKI during follow-up had higher mechanical power (MP) values over the first five days of MV than those who did not develop AKI. In addition, PEEP, peak inspiratory pressure, mean airway pressure, DP, and WOBv were significantly higher in patients who developed AKI, whereas TV and respiratory system compliance did not differ significantly between the groups. The principal finding of this study was that higher mean MP remained independently associated with AKI after adjustment for clinical severity, comorbidities, fluid balance, PEEP, and TV/PBW. Higher mean fluid balance, older age, and trauma were also independently associated with AKI. Importantly, the association between MP and AKI was further supported by sensitivity analyses adjusting for DP and PEEP and by the first-24-h analysis restricted to patients without AKI during the first 24 h. Together, these findings suggest that MP may capture an integrated ventilatory mechanical burden associated with AKI beyond individual ventilatory parameters. Nevertheless, given the observational design and potential residual confounding, these findings should be interpreted as associations rather than evidence of a direct causal renal effect of MP.

Studies have shown that MV is associated with an increased risk of AKI [17,18]. The mechanisms underlying MV-associated AKI are explained by the hemodynamic effects of positive pressure, the effects of arterial blood gas tensions on the renal vasculature, and the inflammatory response [8]. Renal blood flow is influenced by the hemodynamic effects of MV, both directly through changes in cardiac output and through neurohumoral pathways. Increased intrathoracic and right atrial pressures may raise renal venous pressure, thereby reducing the renal perfusion gradient and adversely affecting kidney function through venous congestion [8,19,20]. In our study, similar MAP values between the groups and the lack of central venous pressure (CVP) data limited our ability to directly assess renal perfusion pressure. Fluid balance also emerged as an important factor associated with AKI. In the multivariable analysis, each 1-L increase in mean fluid balance was associated with higher odds of AKI. Fluid accumulation may reflect greater hemodynamic instability, disease severity, and the intensity of resuscitative treatment. Conversely, impaired renal function itself may also contribute to progressive fluid accumulation. Therefore, the relationship between fluid balance and AKI is likely to be bidirectional and should not be interpreted as evidence that positive fluid balance directly caused AKI. Importantly, the association between mean MP and AKI persisted after adjustment for mean fluid balance, indicating that the observed association was not explained solely by differences in fluid status. Nevertheless, the persistence of the association between MP and AKI despite comparable MAP values suggests that, in addition to hemodynamic mechanisms, other pathways may also contribute to the relationship between MV and AKI. These pathways may include the effects of hypoxemia and hypercapnia on intrarenal vascular resistance, which may impair renal perfusion and reduce glomerular filtration. In addition, inflammatory mediators released into the systemic circulation during ventilator-induced lung injury may contribute to renal microcirculatory and tubular epithelial injury [5,7,8,17]. From the perspective of MP, greater mechanical energy transfer to the respiratory system may intensify both hemodynamic stress and ventilator-associated biotrauma, thereby providing a plausible mechanistic link between higher MP and renal injury. These effects may also interact with other determinants of renal perfusion, including fluid status and vasopressor requirements. In our study, the presence of higher urea and creatinine levels, a more positive fluid balance, lower pH and HCO_3_^−^ levels, and higher PaCO_2_ values in the AKI group, together with higher MP, supports this hypothesis. The differences in procalcitonin levels observed during follow-up may reflect differences in inflammatory burden in the AKI group; however, this finding should not be interpreted causally, as renal clearance may influence procalcitonin levels. The potential contribution of hypercapnia to AKI through its effects on the renal vasculature is also consistent with the significantly higher PaCO_2_ levels observed in the AKI group in our study. These findings suggest that the association between MP and AKI may not be explained solely by systemic hypotension and that increased mechanical stress, venous congestion, impaired gas exchange, and metabolic disturbances may collectively contribute to this relationship.

Importantly, MP should be interpreted in this context from several perspectives. First, MP is an integrated measure of the mechanical energy transferred from the ventilator to the respiratory system. Second, higher MP may also reflect greater underlying respiratory and overall disease severity, as patients with more severe illness may require greater ventilatory support. Therefore, although MP represents a potentially modifiable ventilatory exposure, the observational design of our study does not establish a causal relationship between MP and AKI. Whether reducing MP itself can decrease the risk of AKI requires evaluation in prospective interventional studies.

The coexistence of respiratory failure and AKI complicates the management of critically ill patients and is associated with adverse clinical outcomes. Accordingly, careful consideration of lung–kidney interactions is essential in the management of these patients [21].

The independent association between MP and AKI observed in our study supports the findings of recent experimental studies. Similarly, a meta-analysis evaluating the association between IMV and AKI in critically ill patients found that although MV was associated with an increased risk of AKI, neither TV nor PEEP was associated with AKI [6]. In another study involving patients with acute respiratory distress syndrome (ARDS), DP was found to be associated with the development of late-onset AKI [22]. A secondary analysis by Gattarello et al. evaluated the association between 48 h of exposure to high MP and AKI development. MP itself was not independently associated with AKI; however, the PEEP component of MP showed the strongest association with AKI. In the same study, cardiac output, mean perfusion pressure, and CVP were identified as the hemodynamic variables most closely associated with AKI [15]. However, clinical evidence directly evaluating the association between MP and AKI remains very limited. In our study, five-day mean MP was associated with AKI independently of PEEP, TV, disease severity, and other clinical variables. This finding supports the clinical relevance of previous experimental observations and suggests that the total energy load transferred from the ventilator to the respiratory system may capture ventilatory mechanical burden associated with AKI more comprehensively than individual ventilatory parameters. Furthermore, the moderate discriminative performance of mean MP for AKI and the identified cutoff value of 10.38 J/min indicate that MP alone is not sufficient for stand-alone clinical decision-making. Rather than being used as a single trigger, MP should be interpreted as one component of a broader clinical risk assessment incorporating patient characteristics, disease severity, renal function, hemodynamic status, and other relevant clinical factors. In addition, five-day mean MP showed statistically significant but limited discriminative performance for CRRT use (AUC = 0.624). Although this finding suggests an association between MP and CRRT use, the limited discriminative performance indicates that MP alone is insufficient to distinguish patients who received CRRT. AKI in critically ill patients is a multifactorial syndrome resulting from the interplay of numerous factors, including sepsis, hypotension, nephrotoxic drug exposure, comorbidities, and systemic inflammation [7,8,23]. Therefore, the limited discriminative ability of MP when used alone is not unexpected. Nevertheless, the ability to routinely calculate MP during MV without additional cost or invasive procedures may enhance its potential clinical relevance. In particular, future studies should determine whether MP adds incremental value to established clinical risk models for AKI.

One of the notable findings of our study was that MP and DP were higher in the AKI group despite the absence of a significant difference in TV between the groups. Similarly, a meta-analysis evaluating the association between IMV and AKI showed that MV was associated with an increased risk of AKI, whereas no significant association was observed between TV and AKI [6]. This finding may be attributable to the widespread implementation of lung-protective ventilation strategies, which has resulted in relatively standardized TVs in contemporary clinical practice [24]. Therefore, TV alone may not adequately reflect the overall biomechanical load imposed by MV. In contrast, PEEP and DP were consistently higher in patients who developed AKI, while RR became higher later during the observation period. Each of these parameters contributes to MP. Higher airway pressures, particularly through increased intrathoracic pressure, may adversely affect renal perfusion by reducing venous return and cardiac output and by increasing renal venous pressure and congestion. In addition, a higher RR increases the frequency with which mechanical energy is delivered to the respiratory system and may therefore increase cumulative ventilatory energy exposure even when TV remains relatively unchanged. These findings may help explain why MP, which integrates these components, was more strongly associated with AKI than individual ventilatory parameters in our cohort.

The persistently higher MP observed over five consecutive days in patients who developed AKI and its association with AKI throughout the observation period support the need for continuous and dynamic assessment of ventilation strategies in critically ill patients requiring IMV. In this regard, the use of repeated measurements rather than measurements obtained at a single time point represents an important aspect of our study. However, given the observational design, it cannot be concluded that sustained exposure to higher MP directly causes AKI. Importantly, in the sensitivity analysis excluding patients who developed AKI within the first 24 h, first-24-h MP was significantly higher among patients who subsequently developed AKI, suggesting that the observed association may already be present before AKI onset. However, this finding does not establish a predictive or causal relationship. Patients who developed AKI may have required greater ventilatory support because of more severe systemic illness, and higher MP may therefore represent a marker of disease severity rather than a causal factor. Indeed, despite similar APACHE II and SOFA scores, the higher mean airway pressures and FiO_2_ requirements observed in the AKI group may reflect more severe pulmonary dysfunction. This underlying pulmonary severity may represent a common marker associated with both greater ventilatory support requirements and the development of AKI. Furthermore, the MP cutoff of 10.38 J/min identified in our study was lower than the 17 J/min threshold above which Serpa Neto et al. reported a consistent increase in mortality risk [13]. However, these thresholds were derived for different clinical outcomes, and our lower cutoff should not be interpreted as a safety threshold for ventilation. Rather, it may suggest that the MP level associated with renal risk differs from thresholds previously associated with mortality. Importantly, it should not be applied as a rigid cutoff in clinical practice until validated in independent cohorts.

The significantly higher 28-day and ICU mortality rates observed in patients who developed AKI are consistent with previous epidemiological studies [1,23]. The higher prevalence of AKI and more frequent CRRT use during the five-day observation period among patients who died further underscore the association between greater renal disease burden and adverse outcomes. These findings further emphasize the clinical relevance of both AKI occurrence and severity in critically ill patients. The shorter duration of MV and ICU LOS observed in the AKI group may initially appear counterintuitive. Given the substantially higher mortality in this group, earlier death may have truncated both MV duration and ICU LOS; therefore, these shorter durations should not be interpreted as indicating more rapid clinical recovery. This finding highlights the importance of accounting for mortality when interpreting time-dependent clinical outcomes.

This study has several strengths. First, it was conducted in a large cohort of 924 critically ill patients receiving IMV. Data were electronically extracted from the clinical decision support system using structured queries. This approach minimized data loss and reduced the potential for human error, thereby enhancing the reliability of the study data. Second, MV parameters were repeatedly assessed over the first five consecutive days of ventilation rather than at a single time point, allowing the dynamic nature of ventilatory exposure to be captured. In addition, MP was not evaluated in isolation but was analyzed alongside TV, PEEP, DP, respiratory system compliance, and other ventilatory parameters, enabling a comparative assessment of different components of ventilatory exposure. Finally, multivariable regression and ROC analyses were used to evaluate both the independent association of MP with AKI and its potential clinical utility.

This study also has several important limitations. The five-day mean MP in some patients who developed AKI included ventilatory measurements obtained after AKI onset. Therefore, the temporal direction of the association between MP and AKI could not be established, and the observed findings should not be interpreted as evidence of a predictive or causal relationship. The lack of data on intravenous fluids and diuretic therapy administered before ICU admission may have influenced urine output measured during the ICU stay and, consequently, AKI classification. Detailed indications for CRRT initiation and the timing of CRRT initiation relative to the five-day MP assessment period were not available for analysis. Therefore, the temporal relationship between MP exposure and CRRT initiation could not be established. In patients receiving CRRT, post-CRRT serum creatinine and urea levels were influenced by extracorporeal solute clearance and were not censored according to CRRT initiation. Therefore, their longitudinal trajectories cannot be interpreted as reflecting the natural course of renal function alone. In addition, death represents a competing event for MV duration and ICU length of stay. Therefore, the shorter durations observed in the AKI group may reflect truncation by earlier mortality rather than faster clinical recovery. Additionally, owing to the retrospective design, longitudinal data on sedation depth (e.g., Richmond Agitation-Sedation Scale (RASS) scores), quantitative measures of spontaneous respiratory effort, and neuromuscular blockade use were not systematically available. Therefore, active and passive breathing conditions could not be fully distinguished throughout the five-day observation period, which should be considered when interpreting the calculated MP values. Despite adjustment for measured clinical severity, ventilatory variables, and fluid balance, residual confounding cannot be excluded. Several potentially relevant respiratory, hemodynamic, and treatment-related factors were not consistently available for the entire cohort. These included detailed vasopressor exposure, fluid administration, nephrotoxic and antimicrobial exposure, contrast exposure, cardiac function and venous congestion, longitudinal lactate and shock status, surgical status, specific causes of AKI, detailed oxygenation indices, prone positioning, and recruitment maneuvers. Therefore, the observed association between MP and AKI may partly reflect greater respiratory and systemic illness severity rather than a direct renal effect of MP. The requirement for at least five consecutive days of IMV with complete MP data resulted in a highly selected cohort and introduced potential selection and survivorship bias. Patients who were extubated, died, had interruption of IMV, or had incomplete ventilatory data before completion of the observation period were excluded. Accordingly, the findings should not be extrapolated to patients requiring fewer than five days of IMV. In addition, detailed ARDS status and severity according to the Berlin definition were not consistently available for the entire cohort. Finally, the single-center tertiary ICU setting, with its specific case mix and ventilatory practices, may limit the generalizability of the findings to other ICUs and patient populations.

Our findings suggest that MP may represent a physiological variable worthy of further investigation for assessing ventilation-related burden associated with AKI in critically ill patients receiving IMV. However, prospective studies evaluating MP exposure before AKI onset, together with time-dependent analyses, are needed to determine its potential role in AKI risk stratification.

## 5. Conclusions

In critically ill patients receiving IMV, higher mechanical power (MP) was independently associated with AKI after adjustment for measured covariates. The cutoff value of 10.38 J/min demonstrated moderate discriminative performance and should be regarded as a hypothesis-generating finding for future research rather than a definitive clinical threshold. Given the observational design and the temporal overlap between the five-day MP assessment and AKI onset, these findings should not be interpreted as evidence of a predictive or causal relationship. Prospective studies evaluating MP exposure before AKI onset and incorporating time-dependent analyses are needed to further clarify the temporal relationship between MP and AKI.

## Figures and Tables

**Figure 1:**
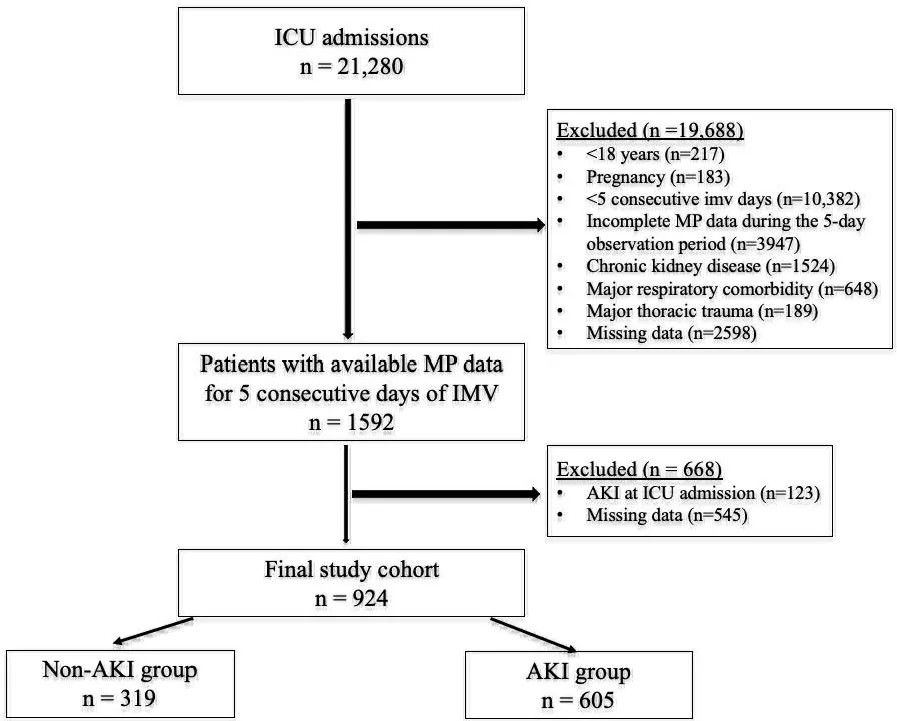
Flow chart of the study population. Abbreviations: ICU, intensive care unit; IMV, invasive mechanical ventilation; AKI, acute kidney injury; MP, mechanical power.

**Figure 2:**
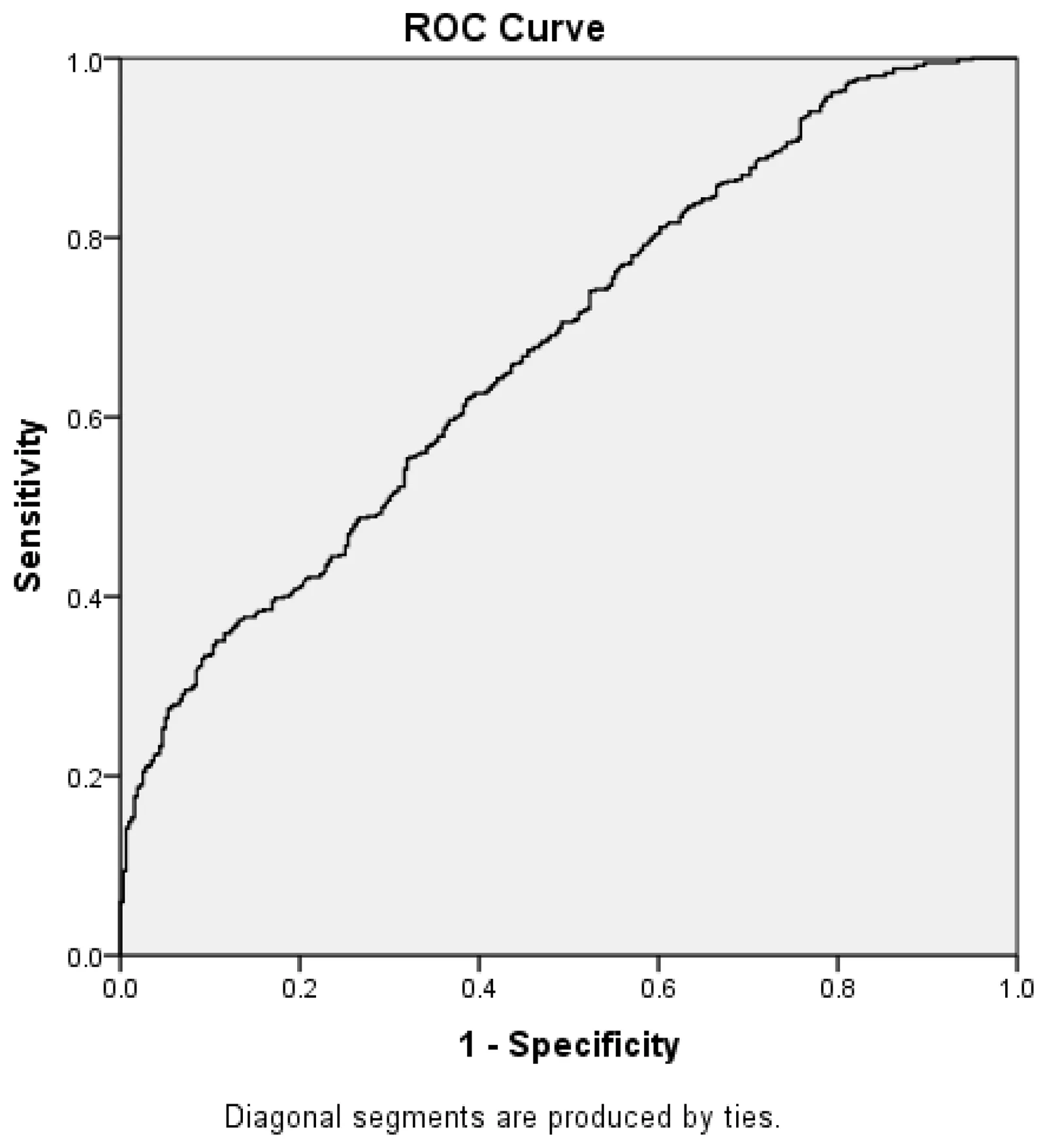
Receiver operating characteristic (ROC) curve of mean mechanical power for discriminating between patients with and without AKI.

**Figure 3:**
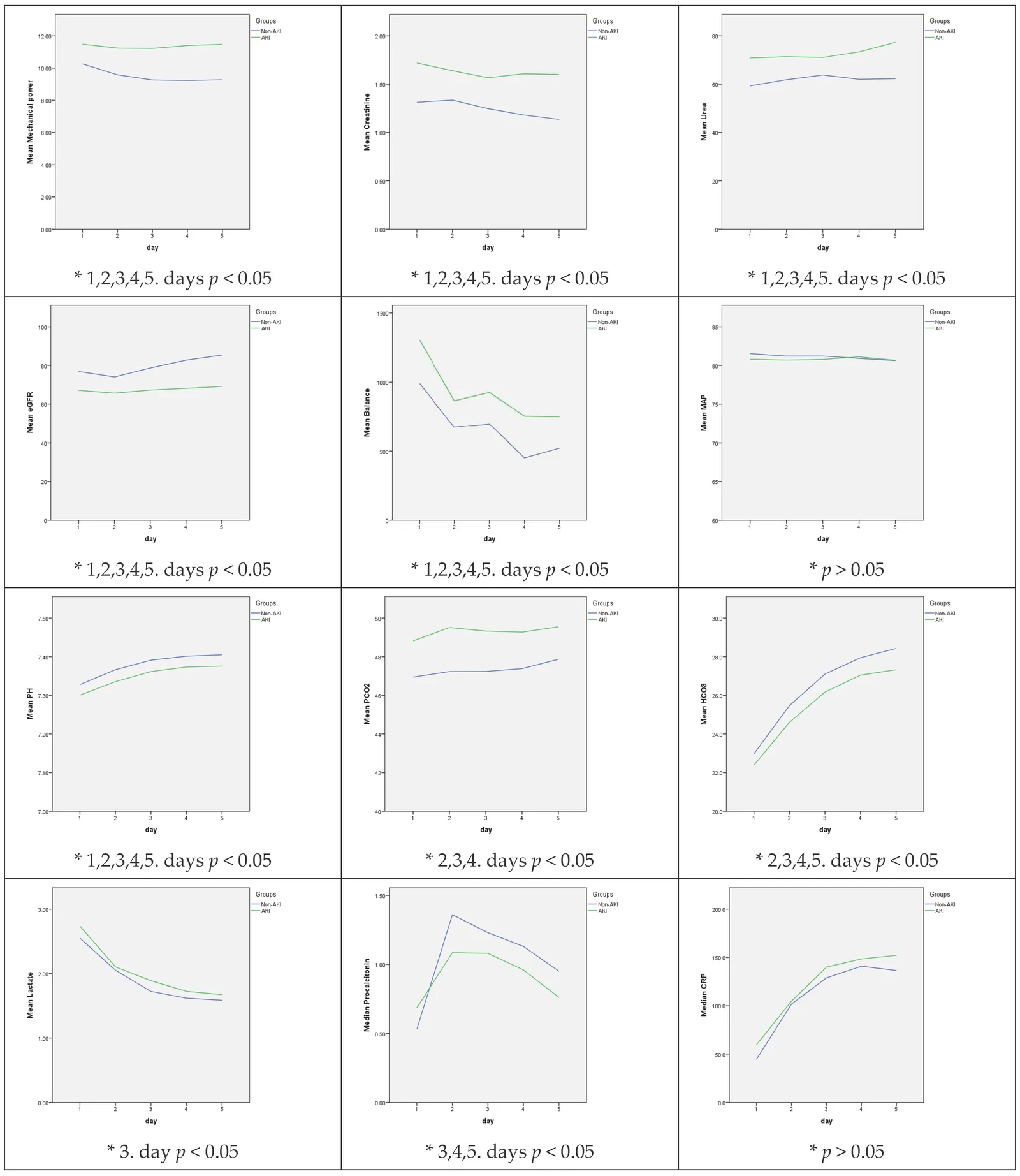
Five-day trajectories of mechanical power, renal, metabolic, inflammatory, and hemodynamic parameters according to AKI status. Explanation: * Normally distributed continuous variables, presented as mean, were compared using the independent-samples *t*-test, whereas non-normally distributed continuous variables, presented as median, were compared using the Mann–Whitney U test. Abbreviations: eGFR, estimated glomerular filtration rate; MAP, mean arterial pressure; PaCO_2_, arterial partial pressure of carbon dioxide; HCO_3_, bicarbonate; CRP, C-reactive protein. Creatinine and urea measurements were not censored after CRRT initiation; therefore, trajectories in the AKI group may reflect both changes in renal function and the effects of CRRT-mediated solute clearance.

**Figure 4:**
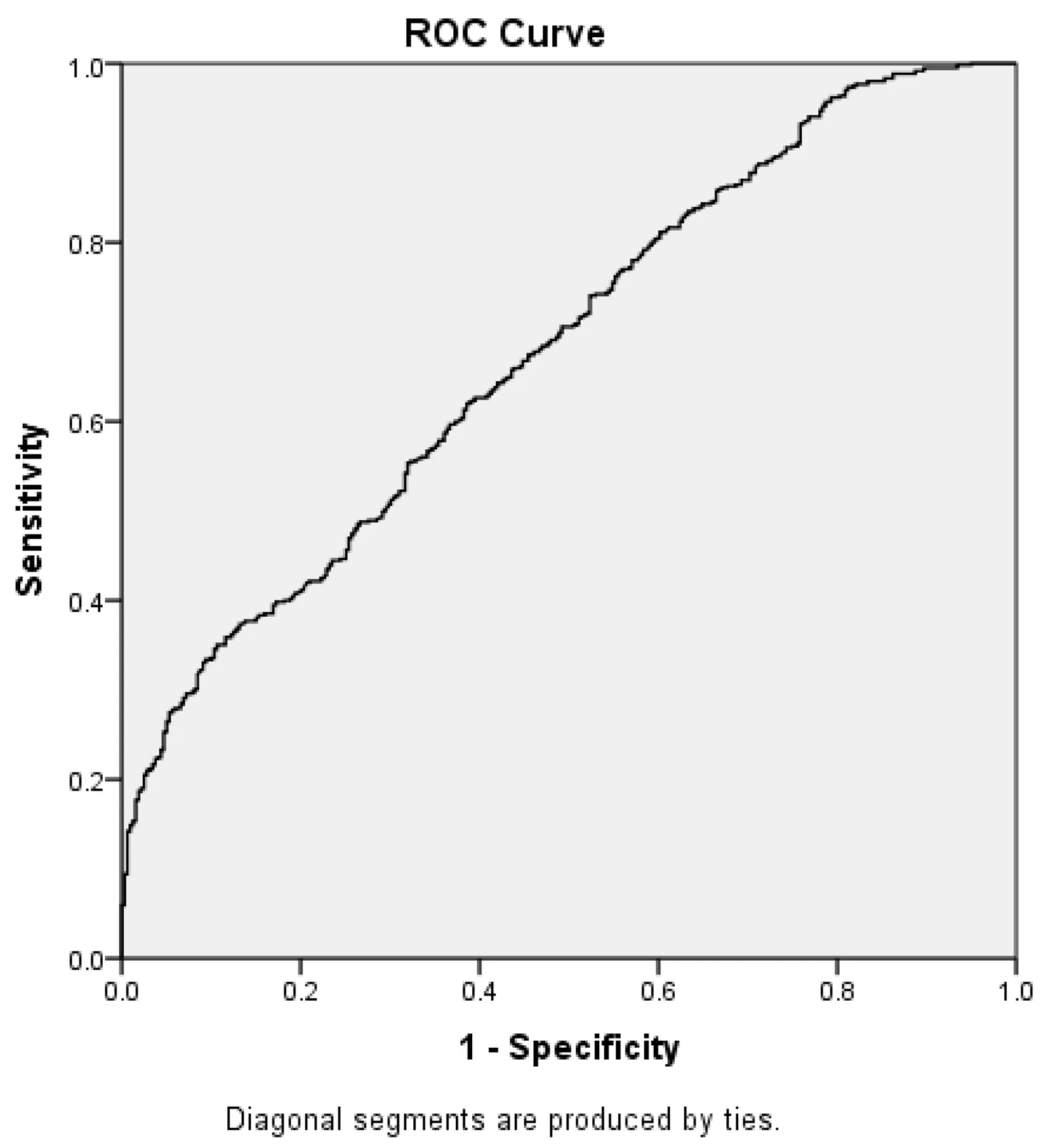
Receiver operating characteristic curve showing the discriminative performance of five-day mean mechanical power for CRRT use.

**Table 1:** Sociodemographic characteristics and admission diagnosis of the patients.

Parameters	Non-AKI *n* = 319 (34.5%)	AKI *n* = 605 (65.5%)	*p* Value
Age (years), mean ± SD	56.88 ± 17.51	58.61 ± 17.81	0.131
BMI (kg/m^2^), mean ± SD	28.62 ± 6.82	28.13 ± 6.66	0.297
APACHE II, median (IQR)	22 (18–28)	24 (19–29)	0.078
SOFA, median (IQR)	10 (8–12)	10 (8–13)	0.150
GCS, median (IQR)	6 (3–11)	6 (3–12)	0.749
Sex			0.372
Female	125 (39.2)	219 (36.2)	
Male	194 (60.8)	386 (63.8)	
Comorbidity	176 (55.2)	354 (58.5)	0.329
Hypertension	81 (25.4)	172 (28.4)	0.325
Diabetes mellitus	51 (16.0)	127 (21.0)	0.067
CVD	53 (16.6)	110 (18.2)	0.552
CAD	42 (13.2)	106 (17.5)	0.086
COPD	25 (7.8)	49 (8.1)	0.889
Malignancy	27 (8.5)	46 (7.6)	0.645
Liver disease	3 (0.9)	14 (2.3)	0.108 *
Dementia	6 (1.9)	18 (3.0)	0.320
Other	16 (5.0)	22 (3.6)	0.315
Admission diagnosis			0.495
CVD	40 (12.5)	72 (11.9)	0.777
Cardiac disease	26 (8.2)	70 (11.6)	0.105
Pulmonary cause of ICU admission	115 (36.1)	224 (37.0)	0.770
Metabolic disease	9 (2.8)	14 (2.3)	0.638
Trauma	36 (11.3)	82 (13.6)	0.326
Sepsis	56 (17.6)	92 (15.2)	0.355
Malignancy	4 (1.3)	10 (1.7)	0.437 *
Postoperative	6 (1.9)	8 (1.3)	0.509
GIB	20 (6.3)	27 (4.5)	0.235
Other	7 (2.2)	6 (1.0)	0.140

Data are presented as mean ± standard deviation (SD), median (interquartile range [IQR]), or *n* (%), as appropriate. Fisher’s exact test was used when the assumptions of the chi-square test were not met. The *p* value presented for admission diagnosis represents the overall comparison between groups. * Fisher exact test. Abbreviations: AKI, acute kidney injury; BMI, body mass index; APACHE II, Acute Physiology and Chronic Health Evaluation II; SOFA, Sequential Organ Failure Assessment; GCS, Glasgow Coma Scale; CVD, cerebrovascular disease; CAD, coronary artery disease; COPD, chronic obstructive pulmonary disease; GIB, gastrointestinal bleeding.

**Table 2:** Ventilatory parameters during the first five days of mechanical ventilation according to AKI status.

Parameter	Non-AKI (*n* = 319; 34.5%)	AKI (*n* = 605; 65.5%)	*p* Value
FiO_2_, %			
Day 1	51.7 ± 13.6	52.4 ± 14.1	0.431
Day 2	45.1 ± 10.8	47.0 ± 11.7	0.015
Day 3	42.4 ± 9.9	45.2 ± 11.6	<0.001
Day 4	41.6 ± 9.2	44.6 ± 11.7	<0.001
Day 5	41.6 ± 9.7	44.6 ± 11.5	<0.001
5-day average	44.3 ± 8.8	46.8 ± 10.2	<0.001
PEEP, cmH_2_O			
Day 1	7.48 ± 2.23	7.86 ± 2.16	0.012
Day 2	7.34 ± 2.09	7.92 ± 2.08	<0.001
Day 3	7.26 ± 2.01	7.81 ± 2.01	<0.001
Day 4	7.19 ± 1.83	7.70 ± 2.04	<0.001
Day 5	7.17 ± 1.85	7.64 ± 1.98	<0.001
5-day average	7.29 ± 1.82	7.78 ± 1.85	<0.001
Peak inspiratory pressure, cmH_2_O			
Day 1	21.5 (17.5–24.9)	22.5 (18.1–26.0)	0.021
Day 2	20.8 (17.6–24.1)	22.3 (18.5–25.7)	<0.001
Day 3	20.2 (17.6–23.2)	22.1 (18.9–25.7)	<0.001
Day 4	20.3 (17.9–23.3)	21.9 (19.1–25.3)	<0.001
Day 5	20.3 (17.5–23.0)	22.0 (19.1–25.3)	<0.001
5-day average	21.0 (17.8–23.3)	22.0 (19.0–25.7)	<0.001
Plateau pressure, cmH_2_O			
Day 1	21.4 (17.6–24.7)	22.5 (18.1–26.0)	0.006
Day 2	20.7 (17.6–24.1)	22.4 (18.7–25.8)	<0.001
Day 3	20.2 (17.6–23.1)	22.3 (19.0–25.5)	<0.001
Day 4	20.2 (17.8–23.2)	21.9 (19.1–25.1)	<0.001
Day 5	20.2 (17.4–22.9)	22.0 (19.2–25.2)	<0.001
5-day average	20.9 (17.8–23.3)	22.1 (19.1–25.7)	<0.001
Mean airway pressure, cmH_2_O			
Day 1	12.8 ± 3.7	13.0 ± 3.4	0.359
Day 2	12.4 ± 3.2	13.2 ± 3.3	0.002
Day 3	12.3 ± 3.0	13.3 ± 3.2	<0.001
Day 4	12.3 ± 2.9	13.3 ± 3.3	<0.001
Day 5	12.3 ± 2.9	13.4 ± 3.2	<0.001
5-day average	12.4 ± 2.9	13.3 ± 3.0	<0.001
Tidal volume, mL/kg			
Day 1	6.91 ± 1.98	7.07 ± 1.32	0.310
Day 2	6.79 ± 2.02	6.99 ± 1.15	0.197
Day 3	6.67 ± 1.97	6.98 ± 1.08	0.044
Day 4	6.70 ± 1.99	6.99 ± 1.09	0.054
Day 5	6.67 ± 2.01	6.95 ± 1.18	0.074
5-day average	6.73 ± 1.92	7.0 ± 0.99	0.081
Respiratory rate, breaths/min			
Day 1	15.0 (13.9–16.3)	15.0 (14.0–16.0)	0.977
Day 2	14.9 (13.5–15.7)	15.0 (13.8–16.0)	0.067
Day 3	14.7 (13.4–15.6)	15.0 (14.0–16.0)	<0.001
Day 4	14.5 (13.6–15.6)	15.1 (14.0–16.2)	<0.001
Day 5	14.8 (13.8–15.7)	15.1 (14.0–16.2)	<0.001
5-day average	14.7 (13.9–15.6)	15.1 (14.2–16.0)	<0.001

Data are presented as mean ± standard deviation (SD) or median (interquartile range [IQR]), as appropriate. The 5-day average represents the average value calculated over the first five days of mechanical ventilation for each patient. Abbreviations: AKI, acute kidney injury; FiO_2_, fraction of inspired oxygen; PEEP, positive end-expiratory pressure.

**Table 3:** Mechanical power, respiratory mechanics, and clinical outcomes according to AKI status.

Parameter	Non-AKI (*n* = 319; 34.5%)	AKI (*n* = 605; 65.5%)	*p* Value
Mechanical power, J/min			
Day 1	10.26 ± 3.14	11.48 ± 3.36	<0.001
Day 2	9.58 ± 2.99	11.23 ± 3.41	<0.001
Day 3	9.26 ± 2.86	11.21 ± 3.26	<0.001
Day 4	9.23 ± 3.04	11.40 ± 3.33	<0.001
Day 5	9.27 ± 3.07	11.48 ± 3.52	<0.001
5-day average	9.48 ± 2.50	11.45 ± 2.88	<0.001
Driving pressure, cmH_2_O			
Day 1	13.95 ± 3.43	14.80 ± 4.05	0.002
Day 2	13.58 ± 3.13	14.73 ± 3.68	<0.001
Day 3	13.32 ± 3.05	14.64 ± 3.45	<0.001
Day 4	13.32 ± 2.94	14.81 ± 3.65	<0.001
Day 5	13.32 ± 3.06	14.86 ± 3.84	<0.001
5-day average	13.51 ± 2.73	14.79 ± 3.27	<0.001
Respiratory system compliance, mL/cmH_2_O			
Day 1	36.9 (27.9–47.4)	36.3 (28.8–46.6)	0.890
Day 2	38.0 (29.2–46.9)	37.0 (29.4–45.6)	0.263
Day 3	37.8 (29.3–46.0)	35.9 (29.2–44.8)	0.226
Day 4	37.0 (30.3–46.2)	36.5 (29.4–44.5)	0.137
Day 5	37.6 (28.6–46.3)	36.5 (29.0–44.3)	0.171
5-day average	38.2 (29.8–46.6)	36.7 (29.6–45.0)	0.451
Ventilator work of breathing (WOBv), J/L			
Day 1	1.14 (0.92–1.32)	1.19 (1.0–1.38)	0.003
Day 2	1.08 (0.94–1.27)	1.18 (1.0–1.34)	<0.001
Day 3	1.07 (0.89–1.27)	1.17 (1.01–1.37)	<0.001
Day 4	1.07 (0.91–1.25)	1.18 (1.04–1.37)	<0.001
Day 5	1.07 (0.91–1.24)	1.20 (1.04–1.35)	<0.001
5-day average	1.08 (0.94–1.26)	1.18 (1.05–1.36)	<0.001
Clinical outcomes			
Duration of mechanical ventilation, days	10.58 (7.54–15.54)	9.91 (6.70–14.31)	0.041
ICU length of stay, days	13.29 (8.75–20.16)	12.04 (7.43–18.41)	0.014
28-day mortality, *n* (%)	107 (33.5)	294 (48.6)	<0.001
ICU mortality, *n* (%)	119 (37.3)	305 (50.4)	<0.001

Data are presented as mean ± standard deviation (SD), median (interquartile range [IQR]), or *n* (%), as appropriate. The 5-day average represents the average value calculated over the first five days of mechanical ventilation for each patient. Abbreviations: AKI, acute kidney injury; ICU, intensive care unit; WOBv, ventilator work of breathing.

**Table 4:** AKI stage distribution and renal outcomes according to 28-day survival status among patients who developed AKI.

Parameters	AKI-Survivor *n* = 311 (51.4%)	AKI Non-Survivor *n* = 294 (48.6%)	*p*-Value
New-onset AKI during Day 1, *n* (%)	143 (46.0)	147 (50.0)	0.323
AKI stage first day			
Non-AKI	168 (54.0)	147 (50.0)	0.608
AKI stage 1	75 (24.1)	71 (24.1)	
AKI stage 2	32 (10.3)	32 (10.9)	
AKI stage 3	36 (11.6)	44 (15.0)	
AKI 2. day, *n* (%)	231 (74.3)	214 (72.8)	0.678
AKI stage 2. day			
Non-AKI	80 (25.7)	80 (27.2)	0.161
AKI stage 1	91 (29.3)	97 (33.0)	
AKI stage 2	76 (24.4)	50 (17.0)	
AKI stage 3	64 (20.6)	67 (22.8)	
AKI 3. day, *n* (%)	236 (75.9)	217 (73.8)	0.556
AKI stage 3. day			
Non-AKI	75 (24.1)	77 (26.2)	<0.001
AKI stage 1	86 (27.7)	86 (29.3)	
AKI stage 2	96 (30.9)	51 (17.3)	
AKI stage 3	54 (17.4)	80 (27.2)	
AKI 4. day, *n* (%)	237 (76.2)	222 (75.5)	0.842
AKI stage 4. day			
Non-AKI	74 (23.8)	72 (24.5)	0.311
AKI stage 1	84 (27.0)	83 (28.2)	
AKI stage 2	78 (25.1)	56 (19.0)	
AKI stage 3	75 (24.1)	83 (28.2)	
AKI 5. day, *n* (%)	267 (85.9)	254 (86.4)	0.847
AKI stage 5. day			
Non-AKI	44 (14.1)	40 (13.6)	0.020
AKI stage 1	116 (37.3)	87 (29.6)	
AKI stage 2	77 (24.8)	64 (21.8)	
AKI stage 3	74 (23.8)	103 (35.0)	
AKI stage on the last day of ICU stay, *n* (%)	176 (56.6)	233 (79.3)	<0.001
Non-AKI	135 (43.4)	61 (20.7)	<0.001
AKI stage 1	118 (37.9)	85 (28.9)	
AKI stage 2	33 (10.6)	64 (21.8)	
AKI stage 3	25 (8.0)	84 (28.6)	
CRRT, *n* (%)	64 (20.6)	119 (40.5)	<0.001

Data are presented as *n* (%). Percentages were calculated within the respective 28-day survival groups. AKI status and stage were determined according to the Kidney Disease: Improving Global Outcomes (KDIGO) criteria. “Last-day AKI” refers to AKI status assessed on the last day of the ICU stay. Day 1 AKI refers to new-onset AKI developing within the first 24 h after ICU admission; patients with AKI clinically diagnosed before or at ICU admission and documented as an ICU admission diagnosis were excluded. CRRT use refers to CRRT administered at any time during the five-day observation period. *p* values for AKI stage represent the overall comparison of stage distributions between survivors and non-survivors. Abbreviations: AKI, acute kidney injury; CRRT, continuous renal replacement therapy; ICU, intensive care unit; KDIGO, Kidney Disease: Improving Global Outcomes.

**Table 5:** Multivariable logistic regression analysis of parameters associated with AKI.

Parameters	OR	95% CI	*p*-Value
Age, per 1-year increase	1.012	1.003–1.022	0.009
APACHE II score, per 1-point increase	0.997	0.977–1.017	0.757
SOFA score, per 1-point increase	1.013	0.970–1.058	0.550
Diabetes mellitus	1.215	0.789–1.871	0.377
Hypertension	0.990	0.669–1.465	0.959
Sepsis	0.813	0.541–1.222	0.319
Trauma	1.775	1.094–2.879	0.020
Mean mechanical power, per 1-J/min increase	1.330	1.235–1.432	<0.001
PEEP, per 1-cmH_2_O increase	0.979	0.887–1.081	0.677
Tidal volume, mL/kg PBW	0.983	0.877–1.102	0.774
Mean fluid balance, per 1-L increase	1.477	1.225–1.780	<0.001

Odds ratios (ORs) and 95% confidence intervals (CIs) were obtained from the multivariable logistic regression analysis. For continuous variables, ORs represent the change in the odds of AKI per one-unit increase in the corresponding variable. Abbreviations: OR, odds ratio; CI, confidence interval; APACHE II, Acute Physiology and Chronic Health Evaluation II; SOFA, Sequential Organ Failure Assessment; PEEP, positive end-expiratory pressure; PBW, predicted body weight.

## Data Availability

The patient-level data used in this study are not publicly available and cannot be directly shared with independent researchers due to institutional policies and applicable data-protection requirements. Requests for data access may be submitted to the corresponding author and are subject to institutional review and approval and applicable data-protection regulations.

## Abbreviations

AKI	acute kidney injury
APACHE II	Acute Physiology and Chronic Health Evaluation II
BMI	body mass index
CAD	coronary artery disease
CKD	chronic kidney disease
COPD	chronic obstructive pulmonary disease
CRP	C-reactive protein
CRRT	continuous renal replacement therapy
CVP	central venous pressure
CVD	cerebrovascular disease
DP	driving pressure
ECMO	extracorporeal membrane oxygenation
eGFR	estimated glomerular filtration rate
FiO_2_	fraction of inspired oxygen
GCS	Glasgow Coma Scale
GIB	gastrointestinal bleeding
HCO_3_^−^	bicarbonate
ICU	intensive care unit
IMV	invasive mechanical ventilation
IQR	interquartile range
KDIGO	Kidney Disease Improving Global Outcomes
LOS	length of stay
MAP	mean arterial pressure
MP	mechanical power
MV	mechanical ventilation
PaCO_2_	arterial partial pressure of carbon dioxide
PEEP	positive end-expiratory pressure
RR	respiratory rate
SD	standard deviation
SOFA	Sequential Organ Failure Assessment
SQL	Structured Query Language
TV	tidal volume
WOBv	ventilator work of breathing
